# Estimating health state utilities in Duchenne muscular dystrophy using the health utilities index and EQ-5D-5L

**DOI:** 10.1186/s41687-023-00671-y

**Published:** 2023-12-15

**Authors:** Ivana F. Audhya, Shelagh M. Szabo, Andrea Bever, Fiona O’Sullivan, Daniel C. Malone, David Feeny, Peter Neumann, Susan T. Iannaccone, P. Jayasinghe, Katherine L. Gooch

**Affiliations:** 1 Sarepta Therapeutics, Inc., Cambridge, MA USA; 2 Broadstreet HEOR, 201-343 Railway St, Vancouver, BC V6A 1A4 Canada; 3 The University of Utah, Salt Lake City, UT USA; 4 McMaster University, Hamilton, ON Canada; 5 Tufts Medical Center, Boston, MA USA; 6 The University of Texas Southwestern, Dallas, TX USA

**Keywords:** EQ-5D, HUI, DMD, Duchenne, Utility

## Abstract

**Background:**

The progression of Duchenne muscular dystrophy (DMD) is characterized by loss of ambulation, respiratory insufficiency, cardiomyopathy, and early mortality. DMD profoundly impacts health-related quality-of-life (HRQoL). However, few health state utility data exist; published utilities tend to be derived from small samples for a limited number of health states and are often based on caregiver-reported patient health status. This study estimated utility values for varied clinical and functional health states in DMD, based on patient-reported health status.

**Methods:**

Individuals with DMD in the US aged 12–40 years completed the EQ-5D (5-level) and Health Utilities Index (HUI) preference-based instruments. Based on responses to a clinical questionnaire, participants self-classified into functional health states according to level of lower and upper limb function, use of respiratory support, and presence of cardiomyopathy. Mean [standard deviation (SD)] utility and EQ-5D visual analogue scale (VAS) scores were estimated according to health state; and median (interquartile range) attribute levels calculated to understand which domains of health are most severely affected in DMD.

**Results:**

Of 63 males with DMD, mean (SD) age was 19.8 (6.1) years and 11 (17.5%) were ambulatory. Mean (SD) utility values were 0.92 (0.08; HUI2), 0.84 (0.20; HUI3), and 0.84 (0.13; EQ-5D) for ambulatory patients without cardiomyopathy (n = 10). For non-ambulatory patients with moderately impaired upper limb function, night and daytime ventilation without cardiomyopathy, mean (SD) utilities were 0.49 (0.07) for the HUI2, 0.16 (0.15) for the HUI3 and 025 (0.14) for the EQ-5D. Mean (SD) VAS scores for the same health states were 91 (9) and 83 (21), respectively. In addition to impairments in mobility/ambulation, and self-care, attributes like usual activities and pain also showed notable effects of DMD.

**Conclusions:**

In DMD, although a relationship between disease progression and HRQoL is observed, there is large variability in utility within functional health states, and across instruments. Utility values for less severe non-ambulatory health states described by level of upper limb function are novel. These utility values, derived based on direct patient feedback rather than from caregiver report, are relevant to individuals of varying functional statuses and augment scarce DMD-specific utility data.

**Supplementary Information:**

The online version contains supplementary material available at 10.1186/s41687-023-00671-y.

## Background

Duchenne muscular dystrophy (DMD) is a rare neuromuscular disease characterized by progressive muscle weakening leading to loss of ambulation, respiratory insufficiency, cardiomyopathy, and premature mortality [1]. As DMD is an X-linked disease, males are primarily affected. As DMD progresses and functional ability diminishes, it profoundly impacts the health-related quality-of-life (HRQoL) of patients with DMD, their caregivers, and families [2–5]. Drivers of HRQoL impact in DMD are multifactorial and include functional changes, such as loss of ambulation or upper limb function; factors such as fatigue, that increase with symptom onset and development; and also the increasing social and emotional implications of DMD progression, all of which impact people’s ability to perform activities of daily living [6–9].

Despite the dramatic impact of DMD on HRQoL, current data on health state utility are limited. Health state utilities document individual preferences for the HRQoL impact of a given health state by assigning a value to that health state on a scale of 1 (full health) to 0 (dead) [10]. Values below 0 may occur that reflect health states considered worse than being dead [10]. Utility values are required inputs into many quality-adjusted life-year (QALY)-based economic models, and guidelines for health economic evaluations recommend they be derived from generic instruments such as the Health Utilities Index (HUI) or EuroQoL’s EQ-5D survey [11–13]. These instruments include a HRQoL questionnaire that classifies health status according to a set of pre-defined domains or attributes, and an accompanying formula or set of weights elicited from a sample of the general population for converting responses into utility values. Previous research has identified the domains of ambulation/mobility, self-care, and emotion to be particularly important predictors of change in utility in ambulatory boys with DMD, as they have explained the largest proportion of variability in utility over time [14].

Existing utility value estimates in DMD are limited in that they are derived from a small number of studies and are based on relatively small samples [7, 14, 15]. In addition, utility estimates are most frequently based on classifications of health status provided by caregivers reporting on behalf of patients, rather than directly from patients themselves [3, 4, 16, 17]. Utility values in DMD are also limited in that they predominantly characterize health states by focusing on ambulatory status and fail to address other potentially important aspects of function and HRQoL across the course of the disease. These include upper limb and hand function, mobility, the ability to transfer (into and out of bed, or from the wheelchair to the toilet), and respiratory insufficiency requiring ventilation, which are all important domains contributing to functional status [18] and HRQoL in DMD [6–8]. However utility values reflecting broader aspects of function among those with DMD, for health states that better represent the natural history of this complex and clinically heterogeneous disease, are not presently available. The objective of this study was to estimate EQ-5D-5L and HUI utility values, based on patient-reported health status, for a range of health states representing varied clinical and functional states in DMD.

## Methods

### Participants

Study participants with DMD were recruited through Parent Project Muscular Dystrophy (PPMD), an advocacy group in the United States (US) [19]. An email was sent to the PPMD mailing list informing individuals and families of the study objectives and eligibility criteria. During screening phone calls, study team members determined which household members were interested and eligible to participate. Parents of younger patients often asked their child if they would like to participate and were present during screening. Patients were also informed that they could receive assistance with completing the survey from a parent, other relative, or friend. Eligibility criteria included a confirmed diagnosis of DMD, age between 12 and 40 years, US residency, fluency in English, and capability of providing informed consent. Study participants were remunerated for their time and expertise; and approval for this study was obtained through Advarra institutional review board (IRB).

### Data collection

A survey was developed using a web-based platform to capture self-reported data from study participants. The survey included a clinical questionnaire to document the extent and severity of DMD symptoms, a series of demographic questions to help characterize the study population, as well as a series of validated preference-based measures of HRQoL, including the HUI and EQ-5D (5-level [5L] version) [20, 21].

**Fig. 1:**
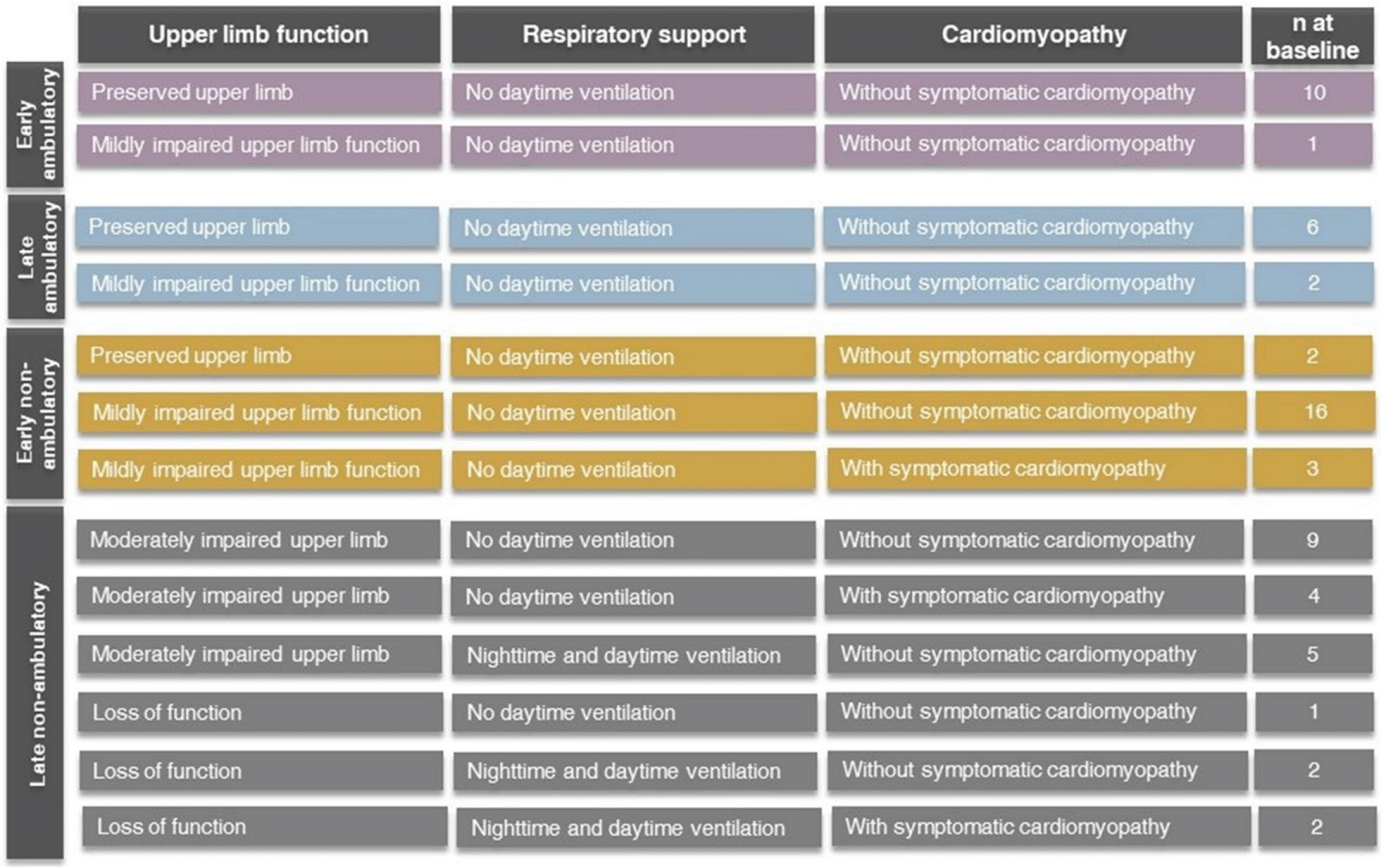
Health states observed among the cohort, by limb function, presence of respiratory support, and cardiomyopathy

The clinical questionnaire was used to understand patient functional health state, based on self-assessment of the primary and secondary manifestations relevant to the natural history of DMD. These included not only lower and upper limb function, but also use of respiratory support for respiratory insufficiency and presence of symptomatic cardiomyopathy, both of which are markers of disease severity. These primary and secondary manifestations have also been deemed important from the patient and caregiver perspective [22, 23]. The questionnaire was based on existing clinical assessments with some modifications [24, 25], which was validated by clinical input (Additional file 1: Appendix Fig. S1) and designed such that patients would be able to self-classify into one of the health states without needing clinical input or assessment. Responses derived from the clinical questionnaire allowed classification to a variety of health states (see Fig. 1), more granular than those previously published which have primarily focused on the ability to walk [3, 4, 14–16].

Because of the relatively small sample size for some health states and for better comparison with existing utility values [7, 14, 15] health states were also assigned based on level of lower and upper limb function alone. Level of lower limb function was used to classify patients as (early) ambulatory, late ambulatory (transitional) or non-ambulatory. Within the non-ambulatory category, patients were further classified as early non-ambulatory if their level of upper limb involvement was considered none or minimal and late non-ambulatory if their level of upper limb involvement was moderate or severe (loss of function).

HRQoL was assessed using responses to the HUI and EQ-5D-5L measures [20, 21]. Responses to HUI questionnaire are used to derive utilities according to two complementary systems: the HUI mark 2 (HUI2) and the HUI mark 3 (HUI3) [21]. The HUI2 descriptive system considers seven attributes: sensation (vision, hearing, and speech), mobility, emotion, cognition, selfcare, pain, and fertility (optional). Levels of impairment for the six relevant HUI2 attributes range from 1 (no impairment) to 4 or 5 (severe impairment). The HUI3 descriptive system considers eight attributes: vision, hearing, speech, ambulation, dexterity, emotion, cognition, and pain. Levels of impairment for the eight HUI3 attributes range from 1 (no impairment) to 5 or 6 (severe impairment).

The EQ-5D (5L) measures health status based on single attributes of mobility, self-care, usual activities, pain/discomfort, and anxiety/depression [20]. Increasing attribute levels indicate worsening function, with a value of 1 representing the best outcome and 5 representing the worst outcome. For example, a score of 5 within the mobility dimension would represent “I am unable to walk”. The EuroQol visual analogue scale (VAS) was also administered, in which participants were asked to represent their current health on the day of completing the survey on a scale from 0 (the worst health you can imagine) to 100 (the best health you can imagine).

Prior to implementation, the survey and online platform were pilot tested for functionality and total completion time. Relevance and acceptability of the demographic and clinical questions to the study population had previously been tested in a qualitative interview study with individuals living with DMD and their caregivers [9].

### Analysis

To estimate individual HUI utility values, as a first step, attribute level codes were derived based on responses to single questions or from combinations of responses to sets of questions. HUI2 and HUI3 attribute levels were transformed using the developers’ algorithm, with the original (Canadian) preference weights applied to HUI responses to estimate multiattribute utility values [26, 27]. Utility values on the HUI3 range from 1.0 to − 0.36, and on the HUI2 range from 1.0 to − 0.03 [26, 27].

To estimate individual EQ-5D utility values, scores for each of the single attributes were compiled into a 5-digit health state. A health state of 11,111, for example, would represent an individual with no problems on any of the five dimensions. These 5-digit health states were then transformed into a single utility index using a US-specific value set, which are weights specific to each of the levels in each dimension [28]. Utility values on the EQ-5D-5L in the US range from 1.0 to − 0.57 [28].

To summarize available utility data, mean (standard deviation [SD]) and median (interquartile range) HUI2, HUI3 and EQ-5D utility values were estimated for health states defined by lower and upper limb function alone as well as by the more granular functional health states that considered use of respiratory support and presence of cardiomyopathy. To understand which attributes were most severely affected, and in turn were most contributing to, observed health state utility in DMD, median (IQR) levels of the attributes of the HUI2, HUI3 and EQ-5D were calculated for each DMD health state. A heat map was generated to visually show the measured values of numerical attribute and domain data using a graded color scheme, to help qualitatively understand which attributes showed the most pronounced impacts in DMD. Mean (SD) EQ-5D VAS scores were also estimated according to patient health state. All analyses were carried out in R (R Core Team, 2022); with EQ-5D analyses performed using the “Eq. 5d” package (V0.10.1, Fraser Morton and Jagtar Singh Nijjar, 2021) [29].

## Results

### Participants

A recruitment email was sent to 2,550 individuals from the PPMD mailing list, and from 64 eligible participants who responded, 63 males with DMD completed the survey. Fifteen participants (23.8%) reported the need for assistance to complete the survey and for 7 of these (46.7%), this assistance was limited to transcribing responses. Mean (SD) participant age was 19.8 (6.1) years; 11 (17.5%) were early ambulatory, 8 (12.7%) were late ambulatory, and 44 (69.8%) were non-ambulatory. Level of upper limb function ranged from preserved (in 18 [28.6%]) to loss of function (in 5 [7.9%]; see Table 1). Nine participants (14.3%) were on night and daytime ventilation at the time of survey, and 9 (14.3%) reported symptomatic cardiomyopathy.

**Table 1:** Demographics and clinical characteristics of participants at baseline

	n = 63
**Mean (SD) participant age, years**	19.8 (6.1)
**Ambulatory status, n (%)**	
Early ambulatory	11 (17.5)
Late ambulatory	8 (12.7)
Non-ambulatory	44 (69.8)
**Upper limb function, n (%)**	
Preserved	18 (28.6)
Mildly impaired	22 (34.9)
Moderately impaired	18 (28.6)
Loss of function	5 (7.9)
**Ventilation use, n (%)**	
No ventilation	30 (47.6)
Nighttime ventilation	24 (38.1)
Daytime ventilation	9 (14.3)
**Cardiomyopathy, n (%)**	
None/none identified	28 (44.4)
Asymptomatic	26 (41.3)
Symptomatic	9 (14.3)

*n* number, *SD* standard deviation

### Utility values

**Fig. 2:**
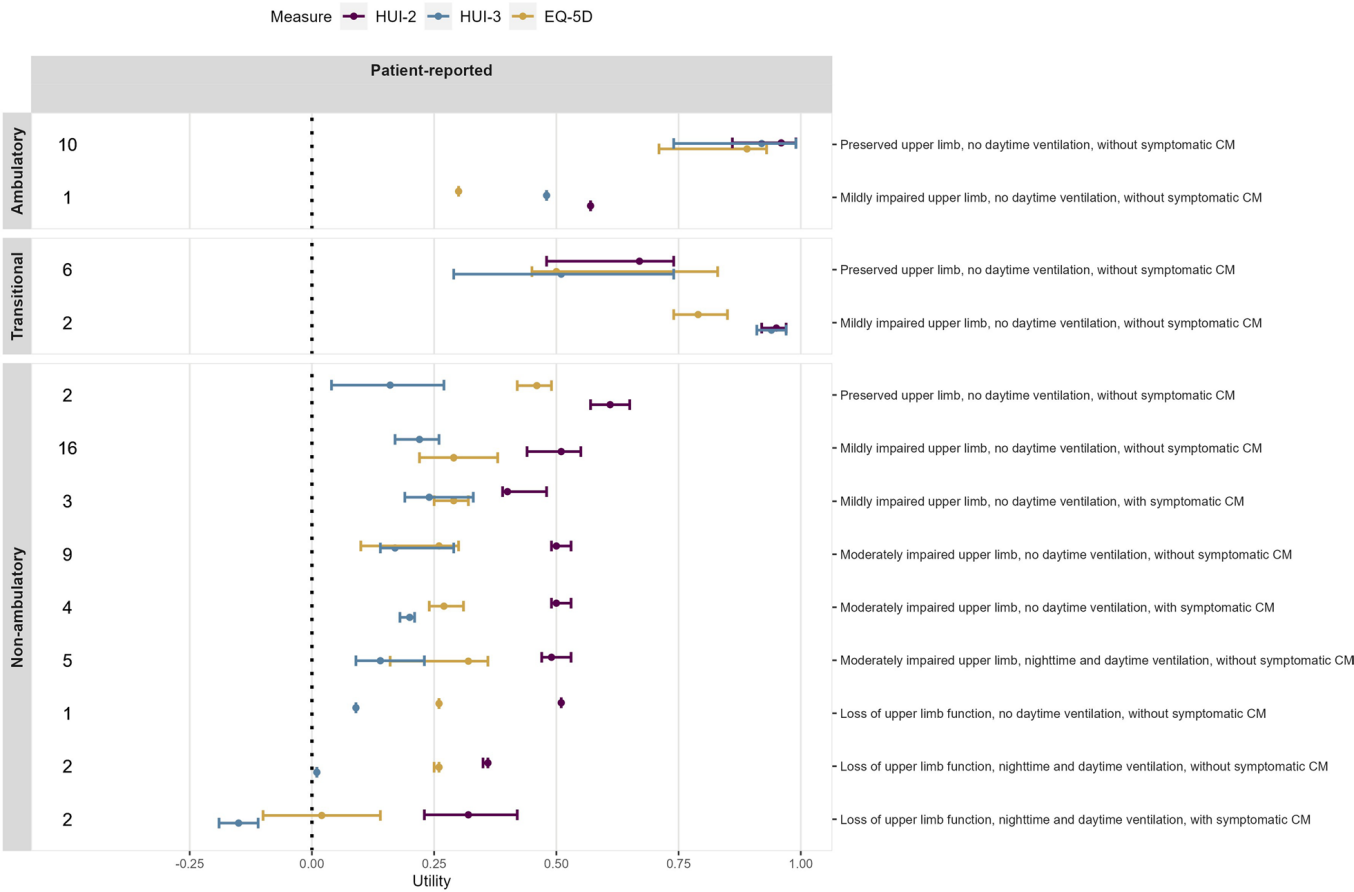
Median (SD) HUI2, HUI3 and EQ-5D utility scores by patient-reported health state

Mean (SD) utility values for early ambulatory participants (n = 11; Table 2) were 0.89 (0.13) for the HUI2, 0.81 (0.22) for the HUI3, and 0.79 (0.20) for the EQ-5D. For late ambulatory participants (n = 8), utility values were considerably lower at 0.71 (0.24), 0.64 (0.32) and 0.64 (0.30), respectively. Mean (SD) utility values for early non-ambulatory participants (n = 21) were 0.49 (0.12) for the HUI2, 0.22 (0.14) for the HUI3, and 0.31 (0.13) for the EQ-5D; for late non-ambulatory participants (n = 23), utility values were 0.47 (0.10), 0.15 (0.15) and 0.22 (0.15), respectively, which is slightly lower than that for early non-ambulatory patients. Median utility values for the same health states (Fig. 2) were slightly higher but with the same relative ordering between instruments.

**Table 2:** Mean (SD) HUI2, HUI3, and EQ-5D utility values per health state

	N	HUI 2	HUI 3	EQ5D-5L
**Early ambulatory**	11	0.89 (0.13)	0.81 (0.22)	0.79 (0.20)
Preserved upper limb, no daytime ventilation, without symptomatic CM	10	0.92 (0.08)	0.84 (0.20)	0.84 (0.13)
Mildly impaired upper limb, no daytime ventilation, without symptomatic CM	1	0.57 (NA)	0.48 (NA)	0.30 (NA)
**Late ambulatory**	8	0.71 (0.24)	0.64 (0.32)	0.64 (0.30)
Transitional, preserved upper limb, no daytime ventilation, without symptomatic CM	6	0.64 (0.22)	0.54 (0.31)	0.59 (0.33)
Transitional, mildly impaired upper limb, no daytime ventilation, without symptomatic CM	2	0.95 (0.07)	0.94 (0.09)	0.79 (0.16)
**Early non-ambulatory**	21	0.49 (0.12)	0.22 (0.14)	0.31 (0.13)
Preserved upper limb, no daytime ventilation, without symptomatic CM	2	0.61 (0.11)	0.16 (0.33)	0.46 (0.10)
Mildly impaired upper limb, no daytime ventilation, without symptomatic CM	16	0.49 (0.12)	0.21 (0.12)	0.30 (0.14)
Mildly impaired upper limb, no daytime ventilation, with symptomatic CM	3	0.45 (0.10)	0.27 (0.14)	0.29 (0.07)
**Late non-ambulatory**	23	0.47 (0.10)	0.15 (0.15)	0.22 (0.15)
Moderately impaired upper limb, no daytime ventilation, without symptomatic CM	9	0.49 (0.05)	0.22 (0.11)	0.22 (0.15)
Moderately impaired upper limb, no daytime ventilation, with symptomatic CM	4	0.52 (0.05)	0.20 (0.02)	0.27 (0.08)
Moderately impaired upper limb, nighttime and daytime ventilation, without symptomatic CM	5	0.49 (0.07)	0.16 (0.15)	0.25 (0.14)
Loss of upper limb function, no daytime ventilation, without symptomatic CM	1	0.51 (NA)	0.09 (NA)	0.26 (NA)
Loss of upper limb function, nighttime and daytime ventilation, without symptomatic CM	2	0.36 (0.01)	0.01 (0.00)	0.26 (0.01)
Loss of upper limb function, nighttime and daytime ventilation, with symptomatic CM	2	0.32 (0.27)	− 0.15 (0.11)	0.02 (0.34)

Considering the health states at the more granular level incorporating use of respiratory support and presence of cardiomyopathy, mean (SD) utility values were 0.92 (0.08) for the HUI2, 0.84 (0.20) for the HUI3, and 0.84 (0.13) for the EQ-5D for the least severe health state including *ambulatory patients with preserved upper limb function without cardiomyopathy or daytime ventilation* (n = 10; Table 2). For the *non-ambulatory with mildly impaired upper limb function, and without daytime ventilation or cardiomyopathy* health state (n = 16*)*, mean utility values were 0.49 (0.12) for the HUI2, 0.21 (0.12) for the HUI3, and 0.30 (0.14) for the EQ-5D. For the most severe health state including *non-ambulatory patients with loss of upper limb function, nighttime and daytime ventilation, and symptomatic cardiomyopathy* (n = 2) mean utility values were 0.32 (0.27) for the HUI2, -0.15 (0.11) for the HUI3, and 0.02 (0.34) for the EQ-5D (Table 2).

### Attributes showing the greatest impact due to DMD

**Fig. 3:**
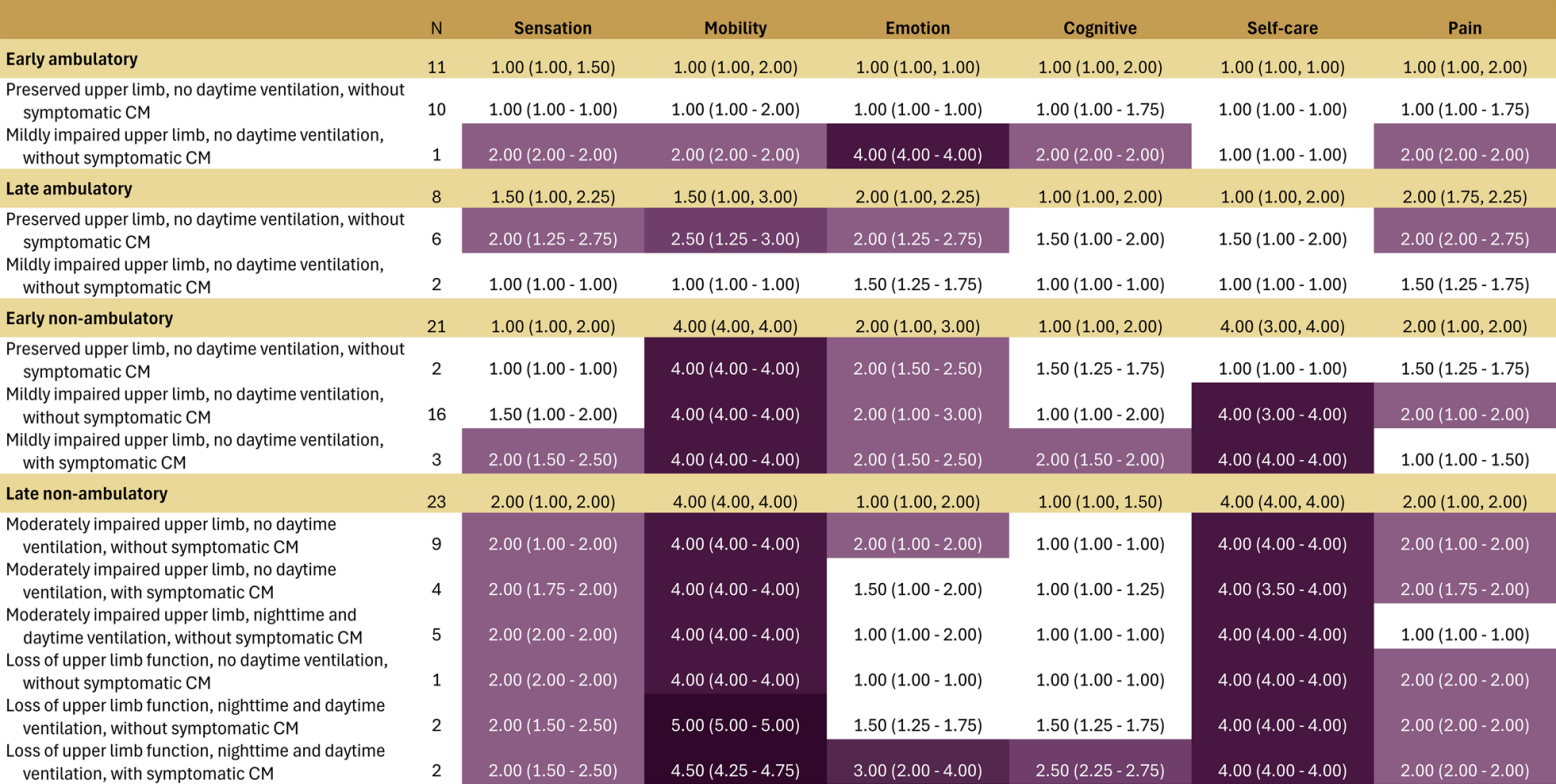
Median (IQR) HUI-2 attribute scores per health state

Heat maps demonstrated that for the HUI2, the attributes with the most pronounced impacts included mobility, followed by self-care, sensation, and pain (Fig. 3). Mobility scores worsened with progressive decline of lower and upper limb function. While median (IQR) self-care scores showed no impairment for ambulatory participants (1.0 [1.0–1.0]; n = 10), severe impairments in self-care were reported for non-ambulatory participants with mild impairments in upper limb function (4.0 [4.0–4.0]; n = 16) through to those with loss of upper limb function, nighttime and daytime ventilation, and symptomatic cardiomyopathy (4.0 [4.0–4.0], n = 2). Lesser impairments were observed for sensation and pain, with median scores ranging between 1 and 2 across health states; and tended to be worse among those in non-ambulatory, vs. ambulatory, health states.

**Fig. 4:**
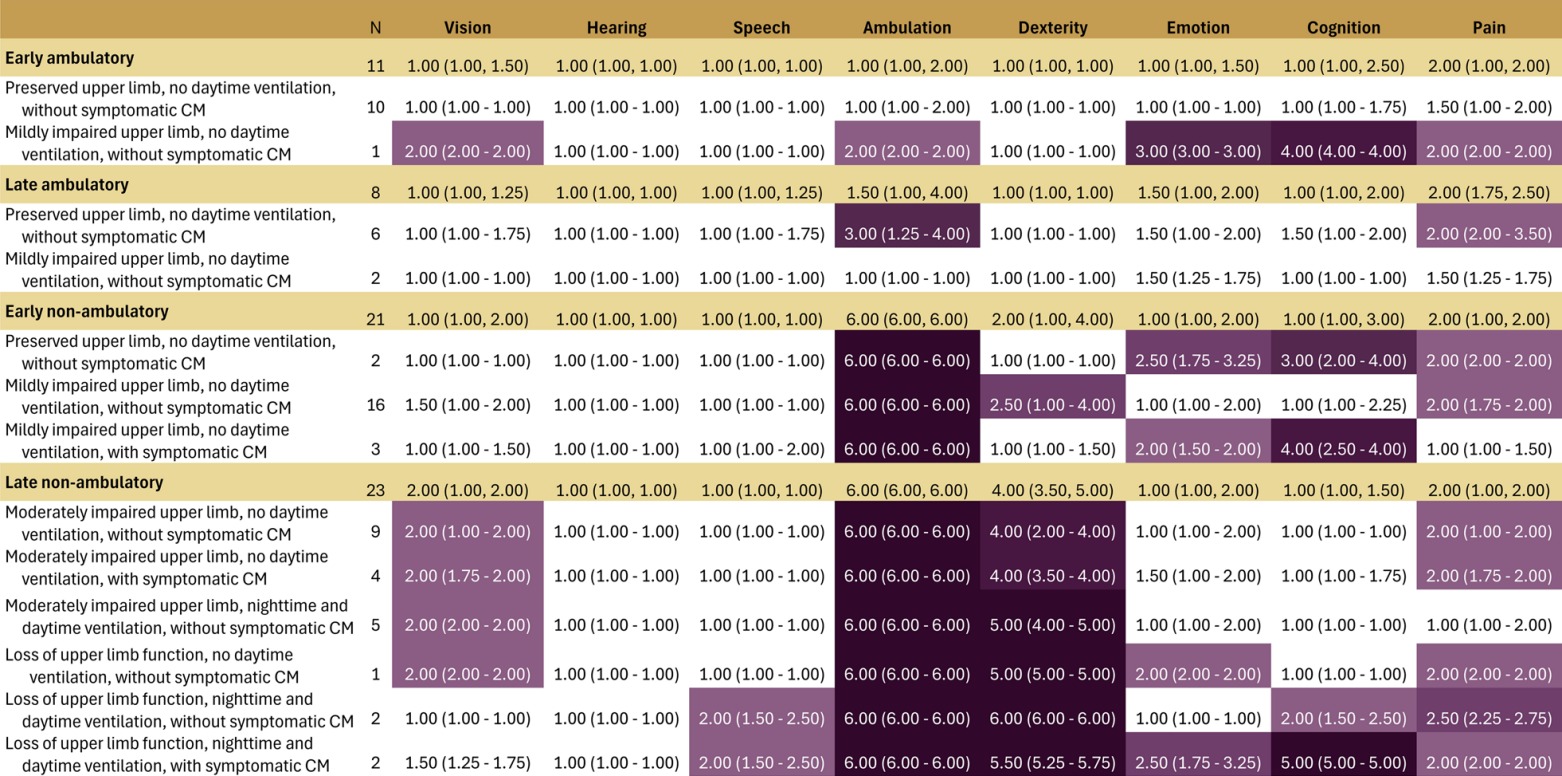
Median (IQR) HUI-3 attribute scores per health state

For the HUI3, the attributes with the most pronounced impacts included ambulation, followed by dexterity, pain, emotion, and to a lesser extent vision and cognition (Fig. 4). Ambulation scores also worsened with increases in lower and upper limb impairment. For dexterity, scores showed little impairment among those in ambulatory through early non-ambulatory health states, with a pronounced impact notable for health states involving moderate impairments in upper limb function (median dexterity score, 4.0) or loss of upper limb function (median dexterity score, 5.0–6.0). The impact of pain and emotion as measured by the HUI3 fluctuated (median scores ranging from 1–2.5) across health states rather than increasing consistently with increasing functional impairment in DMD.

**Fig. 5:**
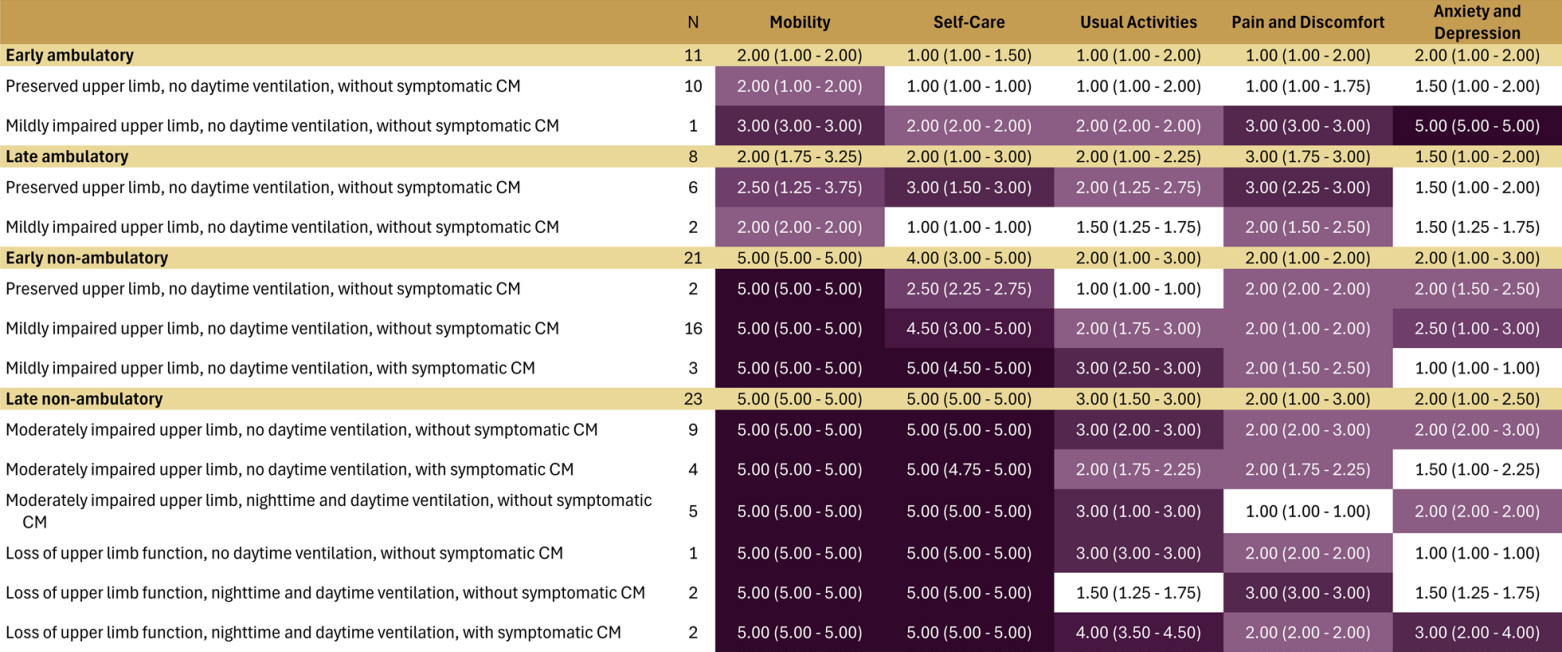
Median (IQR) EQ-5D attribute scores per health state

For the EQ-5D, the attributes with the most pronounced impacts included were mobility, followed by self-care, usual activities, pain/discomfort and anxiety/depression (Fig. 5). Mobility and self-care scores showed mild to moderate impairments (median scores, 1–3) among ambulatory or transitional participants; for non-ambulatory participants with any upper limb impairment, scores were uniformly poor (median, 4.5–5). Scores for usual activities showed mild impairments (median scores, 1–2) among ambulatory or transitional participants and generally increased with increasing health state severity. However even among the most progressed health states, scores on usual activities reflected only mild to moderate impairments (median 2–3). Scores for pain and discomfort reflected mild to moderate impairments and were relatively consistent across health states (median scores, 1–3). Scores for anxiety and depression between health states did not correspond with increasing health state severity.

### VAS scores

**Fig. 6:**
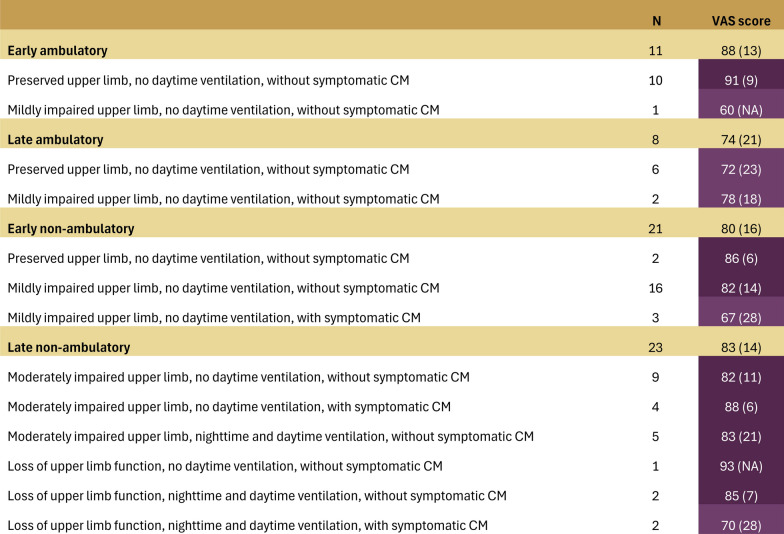
Mean (SD) VAS scores by patient-reported health state

VAS scores tended to be higher than utility values for the same functional health states, and this difference became more pronounced with increasing health state severity (Fig. 6). The mean (SD) VAS score for the *ambulatory with preserved upper limb function without ventilation or cardiomyopathy* health state was 91.0 (9.0) and for the *non-ambulatory with mildly impaired upper limb function, and without ventilation or cardiomyopathy* health state (n = 16), the mean (SD) VAS score was 82.0 (14.0). For the most severe health state *non-ambulatory patients with loss of upper limb function, nighttime and daytime ventilation, and symptomatic cardiomyopathy* (n = 2), the mean (SD) VAS score was 70.0 (28.0).

## Discussion

DMD is a complex disease due to its heterogeneous presentation and numerous primary and secondary manifestations – not only loss of lower and upper limb function, but also respiratory involvement and cardiomyopathy all of which contribute to considerable HRQoL impacts from the caregiver and patient perspectives [8, 22, 30]. Estimates of health state utility are therefore important to document the HRQoL implications of DMD progression. Presently-available utility estimates do not reflect the somewhat linear progression of DMD symptom development; as they are based on a limited number of health states and result in rather large stepwise decrements in utility from ambulatory to non-ambulatory states [7, 14, 15]. Thus, utility estimates for a comprehensive, granular set of health states that accurately represent the natural history of DMD and capture differences in morbidity in a nuanced way, are needed.

This study documented the effects of disease progression and HRQoL impact on utility in DMD, with lower utility values recorded for more severe health states. Utility values for health states involving preserved upper limb function among early- or late-ambulatory patients were consistent with, or higher than, other published estimates. A recent systematic review documented utility values ranging from 0.65 to 0.75 for ambulatory patients with DMD, based on caregivers reporting on behalf of patients [15]. Another recent EQ-5D-based study, by Crossnohere et al., reported utility values ranging from 0.49 to 0.65 for ambulatory DMD health states, and while patients were included in that study, values were not presented stratified by respondent type [31]. In addition, that study used the 3-level (rather than 5-level) version of the EQ-5D, which has well-documented limitations in the language used to describe mobility, and ceiling effects due to the 3 level response options that limit informativity [32]. No other utility data were identified for ambulatory health states that relied on patient report (rather than caregivers reporting on their behalf).

While utility values for less severe non-ambulatory states tended to be higher than published estimates including those identified in the recent systematic review [15, 31], this may be because published health states often lacked stratification by level of upper limb function or other factors important to patient status [15]. Utility values for non-ambulatory health states were 0.26 to 0.31 (EQ-5D) in the study by Crossnohere et al. [31] and ranged from 0.15 (HUI3) to 0.44 (EQ-5D) in the aforementioned systematic review [15]. It is notable that the highest utility estimate for a non-ambulatory state in the systematic review (0.44) was based on feedback directly from Dutch adult patients with DMD (rather than caregivers reporting on their behalf); these adult patients would be expected to have relatively progressed DMD as 95% were on some type of ventilation. However, that study did not report upper limb function when presenting utility estimates, so despite being non-ambulatory, patients may have otherwise been very highly functioning on the dimensions captured by the EQ-5D [16].

As existing utility estimates in DMD are largely derived from caregiver report, the absence of direct patient-reported data represented an important gap [7, 14, 15, 31]. Patients have unique insights on living with their health condition, and caregivers may not necessarily understand the exact extent of the HRQoL impact on their child. For example, research in juvenile arthritis suggests that parents can reliably report on more ‘observable’ contributors to HRQoL (such as functional aspects), but are less able to judge pain, emotional impacts, or psychosocial functioning [33, 34]. Additionally, various decision makers and health technology assessment agencies recommend incorporating HRQoL data directly from patients where possible, rather than from caregivers [13, 35]. Nonetheless, in DMD it is not possible to avoid caregiver report, due to the early age at onset and development status of younger patients [36], and behavioral challenges afflicting some individuals with DMD [37]. Even for studies involving adult patients, many still feature caregiver report [14, 31]. While differences in utility according to source of report has not yet been examined in DMD, in other therapeutic areas caregivers tend to report greater HRQoL impacts (that result in lower utility values) than patients [38, 39].

The ordering of the best to worst functional health state (in terms of associated mean utility value) varied slightly by utility instrument; variability in utility was also observed among individuals within a given functional health state. This variability may potentially be due to several reasons: (1) the small sample sizes for some health states, (2) heterogenous health experiences between individuals, even when health states are described with a high degree of clinical granularity; (3) differences in how the same experiences are captured and scored between instruments. For example, mean utility values from the two patients in the *non-ambulatory, preserved upper limb, without symptomatic cardiomyopathy or daytime ventilation* health state were 0.46 (EQ-5D), 0.61 (HUI2), and 0.16 (HUI3). In these two patients, in addition to the large impact of mobility/ambulation on utility, the particularly low HUI3 utility value was also driven by one patient’s score for cognition. Although the same two survey questions contribute to the HUI2 and HUI3 single attribute utility scores for cognition, scoring algorithms differ giving rise to different scores (0.66 on the HUI3 and 0.93 on the HUI2). Other studies have also noted that differences in the health state classification systems of different instruments result in systematically different utility values, even for people in similar disease-specific health states [40–42]. In the current study, the HUI3 classification system produced the broadest range in utility values for all disease-specific health states, with values higher than the EQ-5D for the least progressed health states but similar or lower for more severe health states. These findings highlight the importance of considering the alignment of an instrument’s descriptive system versus the target condition, when selecting a utility measure. In addition to recent work to understand which aspects of HRQoL are most important from the perspective of patients with DMD and their caregivers (‘Project Hercules') [8, 43], further research will be important to assess which dimensions from widely-used, generic preference-based measures are most appropriate for understanding the HRQoL impacts in DMD.

The analysis of instrument-specific attribute scores, which was intended to qualitatively illustrate which aspects of HRQoL are contributing most to disutility in DMD, highlighted the preponderance of patients reporting deficits in ambulation, mobility, and dexterity throughout all but the least progressed health states. Impacts in these attributes in turn drive deficits in ability for self-care and performance of usual activities. Scores on ambulation, mobility or dexterity drop to very low levels even for those in, for example, late ambulatory or non-ambulatory health states with preserved upper limb function, giving rise to large disutility. This reflects the emphasis members of the general public involved in valuation exercises placed on the negative aspects of ambulation loss, potentially underappreciating the importance of unaffected domains compared to patients as they experience ambulation loss. Patient-provided ratings for other attributes (such as usual care, pain or emotion) demonstrated more variability which could be reflecting the heterogeneous experiences of individuals with DMD, but also how the impact of DMD on emotional status or pain can wax and wane in response to functional changes experienced and accommodation to these [9].

Additional context as to how patients view the impact of DMD is provided by comparing VAS scores (from patients) and utility values (reflecting general public preferences). When preference-based measures are used, disease-specific health states are valued via a hypothetical set of health states based on the EQ-5D or HUI descriptive systems. Although the VAS is not preference-based and cannot provide a utility value, it nonetheless is an intuitive experience-based measure that can incorporate wider notions of HRQoL than, for example, the EQ-5D. While the two scales have different lower anchors (VAS scores anchored by the worst health imaginable, vs. utility values anchored by being dead) [44], non-ambulatory patients in the current study valued their current health using the VAS far better compared to indirect utility estimates of that same health state based on general public preferences. This trend has also been observed in other patient populations when comparing perspectives from patients to those of the general public [45–47].

Strengths of this study include the large sample size of patients with DMD who directly reported on their health status, representing a range of health states with varying clinical and functional considerations of DMD. Utility values were elicited for health states reflecting a spectrum of different functional aspects including upper limb function in addition to lower limb function, both equally important determinants of HRQoL in DMD [14]. Multiple instruments were used to assess utility, including the 5-level version of the EQ-5D whose descriptive system better reflects mobility impacts in DMD than the 3-level version of the EQ-5D [32].

Limitations include that, although the sample size was relatively large for a rare condition like DMD, sample sizes for some granular health states were very small. For this reason, we presented estimates both according to granular level of function, but also for less granular health states defined by ambulatory status and upper limb function only. An important implication of focusing on patient self-reported health status is that the sample was limited to those aged 12 years or older, which is aligned with the minimum age of self-report for the HUI [21]. However, this resulted in the exclusion of younger children with DMD, who may have different health experiences than the sample included here. For example, as many with DMD will have lost ambulation prior to age 12 years [1], those in the non-ambulatory health state within the current study would represent a subset of all non-ambulatory patients with DMD. On the contrary, given that ambulatory teenagers with DMD have more disease experience, insight, and knowledge of their eventual prognosis compared to younger children with DMD, the inclusion of older ambulatory patients with DMD could result in a conservative (or lower) estimate of utility for this health state. The study included eligibility criteria that required participants be capable of completing a survey in English and providing informed consent, which may limit the generalizability of the sample. While allowing participants to receive assistance with the survey may have helped to address this limitation, the presence of another individual recording the survey responses or providing other forms of assistance has the potential to influence the responses provided. There are many factors that could impact utility within a given health state that are difficult to quantify or capture, and these may also contribute to within health state variability. Examples include personal access to social and financial resources, features of the built environment (such as whether sidewalks exist, and if they include wheelchair-friendly features like down ramps leading to the road), as well as personal and psychosocial characteristics of the patient and their family members [9, 14].

## Conclusions

In this study, although utility values tend to decline with increasing health state severity, large variability in utility was observed among individuals within the same health state. Utility values for less severe non-ambulatory health states described by level of upper limb function are novel. These utility values, estimated using two different preference-based measures from a large sample from the US, are based on health status self-reported directly by patients, applying a clinical classification system that does not require external clinical or functional assessment. The classification considers numerous patient-relevant and clinically meaningful DMD manifestations, including level of upper limb function but also presence of symptomatic cardiomyopathy and need for ventilation. This resulted in utility values for health states that more accurately describe morbidity and the clinically-heterogeneous natural history, and are relevant to individuals at various stages of disease progression. Describing utility values from larger samples of patients, understanding how utility in DMD changes over time, and further investigating the determinants of utility scores will be important for better understanding the impact of DMD symptoms on utility and patient HRQoL.

### Supplementary Information

**Additional file 1. Appendix Figure 1**: Schema used to classify functional status among study participants.

## Data Availability

The datasets used and/or analysed during the current study are available from the corresponding author on reasonable request.

## Abbreviations

5L: Five-level (version of the EQ-5D survey)
DMD: Duchenne muscular dystrophy
HRQoL: Health-related quality-of-life
HUI: Health utilities index
IQR: Interquartile range
IRB: Investigational review board
PPMD: Parent project muscular dystrophy
QALY: Quality-adjusted life year
SD: Standard deviation
US: United States
VAS: Visual analogue scale
