# Supplementary material for: Estimating health state utilities in Duchenne muscular dystrophy using the health utilities index and EQ-5D-5L
Source: J Patient Rep Outcomes. 2023 Dec 15;7:132. doi: 10.1186/s41687-023-00671-y (PMC10724100; doi:10.1186/s41687-023-00671-y)
Supplement: Supplementary file 1 — Additional file 1. Appendix Figure 1: Schema used to classify functional status among study participants. [file 41687_2023_671_MOESM1_ESM.docx]

**Appendix figure 1:** Schema used to classify functional status among study participants

| **Functional aspect** | **Description** |
| --- | --- |
| **Ambulatory status^1^** |  |
| Ambulatory | I walk all day. I may or may not use a wheelchair or scooter for long distances on special occasions (on vacation, at an amusement park or the zoo). |
| Transitional | I use a wheelchair or scooter some of the day on most days of the week throughout the year, but I can regularly walk down the hall at home without holding anything. |
| Non-ambulatory | I always use a wheelchair or scooter. I may or may not be able to take a few steps by myself. |
| **Upper limb involvement^2^** |  |
| Preserved | I can raise both arms above my head at the same time, without having to make any adjustments |
| Mildly impaired | I have difficulty raising both arms at the same time or raising a heavy object to my shoulder or eye level  OR  I cannot raise my hands above my head but can bring a full cup to my mouth |
| Moderately impaired | I cannot raise a full cup but can use my hands to hold a pen, pick up a coin, or drive power chair |
| Loss of function | I am unable to pick up or hold objects |
| **Ventilation^3^** |  |
| No daytime ventilation | **Must not select:** Ventilation during the day and night |
| Nighttime and daytime ventilation | **Must select:** Ventilation during the day and night |
| **Cardiomyopathy** |  |
| Without symptomatic cardiomyopathy | I have no symptoms of heart problems and my doctor hasn’t told me about any changes to my heart health  OR  I have no symptoms of heart problems, but I don’t know if my doctor/cardiologist has detected any changes through heart tests  OR  I have no outward symptoms of heart problems, but my doctor/cardiologist has detected changes through heart tests (e.g., changes detected through EKG, echocardiogram, or cardiac MRI) |
| Symptomatic cardiomyopathy | I have symptoms of heart problems, such as heart rate or rhythm changes or signs of a weak heart muscle, and my doctor/cardiologist has detected changes through heart tests  OR  I have symptoms of heart problems, such as heart rate or rhythm changes or signs of a weak heart muscle, but I don’t know if my doctor/cardiologist has detected any changes through heart tests |

^1^Ambulatory status was defined using categories from Linda Lowes’ Lab Ambulatory Status algorithm: Lowes L, Reash N. *Lab Ambulatory Status Algorithm: A new tool to determine ambulatory status*. Abigail Wexner Research Institute; 2019.

^2^Upper limb involvement categories were based on the Performance of Upper Limb scales: Mayhew AG, Coratti G, Mazzone ES, et al. Performance of Upper Limb module for Duchenne muscular dystrophy.

Developmental Medicine & Child Neurology. 2020;62(5):633-639.

^3^Ventilation status was based on a question asking participants to indicate all types of respiratory support used including cough assist, nebulizer, BiPAP, nighttime ventilation, ventilation during the day and night, and other techniques and equipment to help with breathing.
